# Electroacupuncture Improves Myocardial Fibrosis in Association With Intestinal Flora and Metabolic Modulation in Mice

**DOI:** 10.1155/crp/7765150

**Published:** 2025-08-28

**Authors:** Xueyun Que, Feiyu He, Qida He, Jieying Lim, Xiaoshuang Li, Xianjun Meng, Zongbao Yang

**Affiliations:** ^1^School of Medicine, Xiamen University, Xiamen 361102, Fujian, China; ^2^College of Acupuncture and Moxibustion and Tuina, Fujian University of Traditional Chinese Medicine, Fuzhou 350122, Fujian, China; ^3^The First Affiliated Hospital of Xiamen University, Xiamen 361003, Fujian, China

**Keywords:** electroacupuncture, intestinal flora, metabonomics, myocardial fibrosis

## Abstract

**Objective:** This study investigates the reparative effect of electroacupuncture on myocardial fibrosis (MF) in mice and explores its impact on intestinal flora and metabolism profile. This examines an investigation into the biological mechanisms underlying electroacupuncture's efficacy in treating MF in mice.

**Methods:** Twenty-four male Kunming mice (27–34 g) were randomized into three groups: normal control (NC, *n* = 8), MF model (MF, *n* = 8), and electroacupuncture treatment (EA, *n* = 8). MF and EA groups received daily subcutaneous ISO injections (25 mg/kg) at the nape for 5 days; NC mice received saline. The EA group underwent 14 days of EA at PC6 (Neiguan). Cardiac function, intestinal flora, and metabolites were assessed post-treatment.

**Results:** EA significantly reduced cardiac weight index (CWI), collagen volume fraction (CVF), and serum procollagen III N-terminal propeptide (PIIINP) and improved left ventricular ejection fraction (LVEF) and shortening fraction (LVFS) (*p* < 0.05). Gut microbiota analysis revealed distinct composition shifts: EA restored *Bacteroidota* abundance and lowered *Firmicutes*/*Bacteroidota* ratios, resembling NC profiles. Notably, differential bacteria (e.g., *Staphylococcus lentus*, *Xylanophilum*) correlated with PIIINP, CVF, and cardiac function. Metabolomics identified reduced TMAO, phenylalanine, acetone, and lactic acid in EA vs. MF (*p* < 0.05). Negative correlations included *Stricto-1* vs. phenylalanine and *Rodentium* vs. acetone.

**Conclusion:** EA ameliorates ISO-induced MF in mice by modulating gut microbiota structure and metabolic profiles, suggesting a microbiota-metabolite axis mediates its therapeutic effects.

## 1. Introduction

Myocardial fibrosis (MF), a hallmark of cardiovascular diseases (CVDs), arises from diabetes, hypertension, and other metabolic disorders. While renin-angiotensin-aldosterone system (RAAS) agents and calcium channel blockers inhibit MF, their long-term use poses risks [1]. Consequently, a new therapy is needed for MF.

Imbalances in gut bacteria and altered metabolism are linked to CVD. This is summarized by Ahmad in his review as cardiointestinal interferences [2]. The predominant distribution of the intestinal flora phylum level in mice is represented by the *Firmicutes* and *Bacteroidota* groups [3]. Propionate and acetate, as a *Bacteroidota-dependent* metabolin, can affect the heart by regulating the host's blood pressure in the vessel and immunological stress. Meanwhile, butyrate, which is metabolized by *Firmicutes*, has also been implicated in cardiac hypertrophy and MF. Butyrate (a *Firmicutes* metabolite) contributes to cardiac hypertrophy and MF. Furthermore, metabolites derived from the gut microbiota, including trimethylamine N-oxide (TMAO) [4], bile acids [5], and tryptophan [6], have been evidenced in multiple studies to potentially exert a pivotal role in CVD by influencing vascular blood pressure, oxidative stress, the inflammatory response, and cholesterol metabolism [7–10].

Acupuncture is a common treatment in Chinese medicine clinics for a range of illnesses, including MF. The primary focus of this therapeutic approach is dynamic and integrated regulation, which aligns with the impact of intestinal flora and its metabolites on the host organism. Acupuncture in MF affects a range of biological pathways, including neurotransmitter release, oxidative stress, gene expression, autophagy, and nerve conduction [11–15]. Nevertheless, the precise mechanisms remain unclear, and the impact of acupuncture on the microflora of MF is rarely acknowledged. Consequently, this study examined the impact of electroacupuncture on intestinal flora imbalance in MF, with the objective of further elucidating the systems biology mechanism of electroacupuncture intervention in MF.

## 2. Materials and Methods

### 2.1. Animals

Twenty-four male Kunming (KM) mice (body weight: 27–34 g) were obtained from Zhejiang Viton Lihua Laboratory Animal Technology Co., Ltd. (production license: SCXK (Zhejiang) 2019-0001). Using SPSS software, the mice were randomly assigned to three groups based on body weight: the normal control (NC) group (NC, *n* = 8), the model group of MF (MF, *n* = 8), and the electroacupuncture group (EA, *n* = 8). Mice were housed under specific pathogen-free (SPF) conditions at Xiamen University at 22°C–26°C, 60%–70% humidity, with a 12-h light/dark cycle. All mice had ad libitum access to water and were acclimated to the environment for 7 days prior to experiments. This study was approved by the Animal Protection and Ethics Committee of Xiamen University (Approval No.: XMULAC20230021), and all procedures complied with the Guidelines for the Good Treatment of Laboratory Animals.

### 2.2. MF Mice Model

The MF mouse model was established via daily subcutaneous injections of isoproterenol (ISO) at 25 mg/kg [16]. Specifically, the MF and EA groups received ISO injections daily for 5 days (starting on day 1), while the NC group received equivalent volumes of 0.9% saline. Successful model establishment was confirmed by histopathological analysis of Masson-stained cardiac sections, which revealed significant blue collagen deposition.

### 2.3. Electroacupuncture Intervention

The electroacupuncture point Neiguan (PC6) was located on the forelimbs, 2 mm proximal to the radiocarpal joint. Beginning on the day of model induction, mice in the NC and MF groups were restrained in mouse holders for 15 min daily for 14 days without intervention. Mice in the EA group received electroacupuncture at PC6 using a needle (0.16 × 13 mm; Tianyi Acupuncture Equipment Co., Ltd., China) inserted vertically into the acupoint, with a second needle of identical specifications placed 1 mm lateral to PC6 at a nonacupoint location. These needles were connected to a G6805-2 electroacupuncture device (Qingdao XinSheng Industrial Co., Ltd., China) delivering sparse-dense waves (sparse wave: 4 Hz; dense wave: 50 Hz) at 2–4 V. The current intensity was set to 1.0 mA, and treatment was administered for 15 min daily, alternating between left and right acupoints for 14 consecutive days.

### 2.4. Sample Collection

One day after echocardiography, blood was drawn from mice's eye sockets and centrifuged at 3500 rpm at 4°C for 15 min (22,331, Eppendorf AG, Hamburg). After collecting the blood, the chest was opened to remove the intact heart, which was weighed, rinsed in 0.9% NaCl solution, and finally stored in 4% paraformaldehyde solution. At the end of cardiac sampling, the abdominal subcutaneous tissue was separated layer by layer to expose the intestine. Intestinal contents were removed to the Eppendorf.

### 2.5. General Observation

General observation on mice was carried out daily since the experiment started, including water and food intake, mental state, defecation, and coat color, and the mice were weighed every other day.

### 2.6. Rotarod Test

The rotational speed of the rotarod (47,650, Ugo Basile, Italy) was set at 10 rpm and each mouse was subjected to the rotarod test 5 times. The longest and shortest times were removed from the statistical data and the average time of 3 rotations was taken to represent the endurance of the exercise.

### 2.7. Echocardiographic Evaluation

On Day 14, all mice had their fur removed from the abdomen. On Day 15, the mice were maintained under anaesthesia via continuous inhalation of 1.5% isoflurane in order to perform echocardiography. Cardiac brachy axial sections of mice were placed on an ultrasound probe and observed in M mode (MS550D, Visual Sonics Vevo 2100). Parameters included the average left ventricular ejection fraction (LVEF) and left ventricular shortening fraction (LVFS) in 3 periods.

### 2.8. Cardiac Weight Mass Index (CWI) and Histological Analysis

CWI is the ratio of cardiac weight to body weight. After 48 h in 4% paraformaldehyde, cardiac tissues were dehydrated and embedded in paraffin. All cardiac tissues were cut into 5 μm samples and stained with Masson (Servicebio, G1006) and HE. An optical microscope (Leica Aperio Versa 200) was used to observe and scan all slices to ascertain the presence and distribution of collagen deposition. The Image-Pro Plus 6.0 software was used to analyze staining signals and calculate the cardiac collagen volume fraction (CVF, %).

### 2.9. Detection of Troponin I Type (cTnl) and procollagen III N-terminal propeptide (PIIINP) Levels

All serum samples were centrifuged (Eppendorf AG, 22,331 Hamburg) at 4°C at 3000 rpm for 10 min, and the supernatant was collected. The operation process was carried out according to the Mice N-Terminal Procollagen III Propeptide enzyme-linked immunosorbent assay (ELISA) Kit (PIIINP) instruction book (Hua-mei Biologe, CSB-E13334m) and ELISA Kit for cTnl (Uscn, SEA478Mu). Using the ELISA detector (BioTeK, Epoch), the OD value was tested after the reaction termination. Subsequently, the standard curve was plotted according to the standard concentration, and the concentration of all samples was calculated.

### 2.10. 16S Ribosomal DNA Analysis

Using a DNA kit (E.Z.N.A. Soil DNA Kit, Omega BioTek, USA), the total DNA was extracted from mice feces. A PCR amplification was performed for the 16S V3-V4 zone, and the criteria are as follows: predegeneration was performed under the condition of 95°C; 30 circles of degeneration, annealing, and radiation in sequence at 95°C for 30 s, 55°C for 30 s, and 72°C for 45 s, and then fixed radiation was performed at 72°C for 10 min. The amplified PCR products were detected by 2% agarose gel electrophoresis, purified using the AxyPrep DNA Gel Extraction Kit, and quantitative tests were performed using a Quantus Fluorometer. Using the TruSeqTM DNA Sample Prep Kit, the database was set up, and a sequence was performed using the MiSeq Reagent Kit (Illumina Co., Ltd.).

All raw sequence data were performed for quality control optimization, and all sample series were randomly selected to the same sequence according to the minimum number of sample sequences. Using UPARS (http://drive5.com/uparse/, version 7.1), the nonrepetitive sequence was performed, and an OTU was performed with a 97% similarity. The notes on taxonomy of species with 70% confidence were performed in Silva 16S rRNA Gene Data (v138) according to the sequences and the corresponding OTUs. The subsequent data were analyzed and plotted on the Mei-ji Biology Cloud Platform (https://cloud.majorbio.com).

### 2.11. Mice Serum Metabolomics Assay

The buffer should be 100% D_2_O: 100 mL D_2_O, 0.633 g K_2_HPO_4_, 0.110 g NaH_2_PO_4_, 1.250 mL internal standard solution. The serum was melted, vortexed, and centrifuged. Mix 150 μL of the upper layer of serum with 450 μL of PBS and vortex for 1 min. 150 μL of the upper serum layer was vortexed with 450 μL of PBS for 1 min to facilitate extraction. The serum sample was mixed with PBS and centrifuged for 10 minutes at 4°C and 16,900 rcf. 550 μL of the sample was transferred to a 5 mm NMR tube. It was protected from light and taken to the MRI lab for testing. The instrumental program settings were as follows: a Bruker AVANCE-III 600 MHz spectrometer was used, with the N_1_ blood pulse, H_2_O + D_2_O with salt solvent, 1000 scans, and the acquisition of spectrograms completed at 293 K [17].

### 2.12. Statistical Analysis

Body weight, CWI, EF, FS, ELISA, and CVF data were analyzed using IBM SPSS Statistics 23.0, and statistics and results were plotted using GraphPad Prism 8.0.1 to graph data and identify differences. Indicators whose measurements conformed to the normal distribution were statistically described by comparing means (independent samples *t* test was chosen for comparisons between two groups; one-way ANOVA was used between multiple groups, in which post hoc tests for multiple comparisons were performed, least significant difference (LDS) was chosen for data that conformed to analysis of variance and Games–Howell was chosen for those that did not conform to the test of ANOVA); and indicators not conforming to the normal distribution were statistically analyzed using the nonparametric rank sum test for statistical analysis (Mann–Whitney U for two-group comparisons and Kruskal–Wallis test for multiple comparisons). The hypothesis test was set at *p* < 0.05, indicating that the difference was statistically significant.

## 3. Results

### 3.1. Regulation of EA on CWI

**Figure 1:**
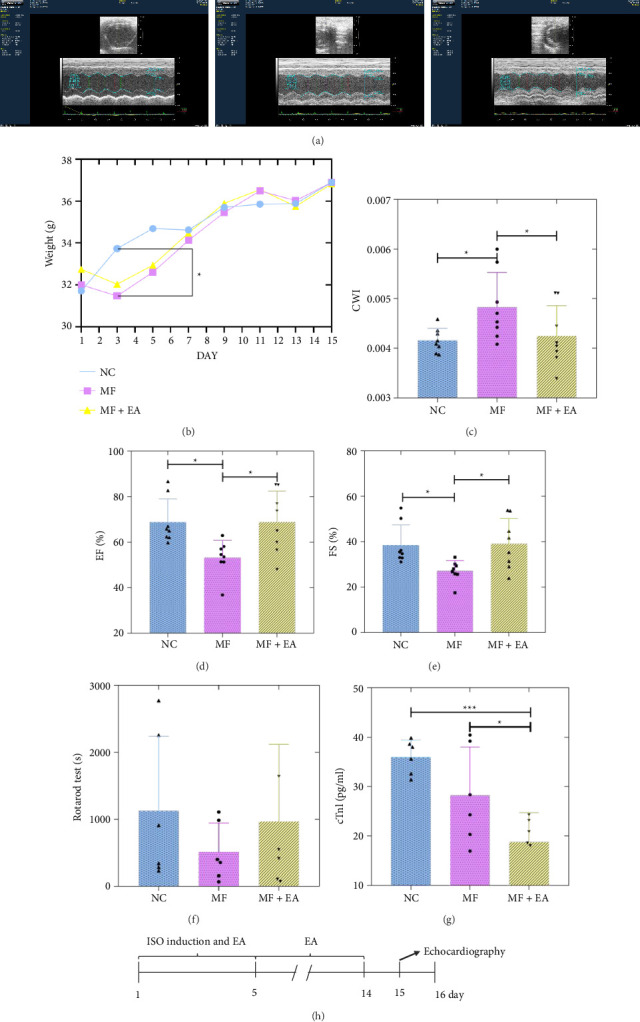
Electroacupuncture improves the general condition of MF. (a) Ultrasonic cardiogram of the heart of the mouse. (b, c) CWI and weight increase trend of mice (*n* = 8). (d, e) EF and FS (*n* = 8). (f) The rotarod test of mice(*n* = 6). (g) cTnl of mice (*n* = 6). (h) Experimental design ^∗^*p* < 0.05, ^∗∗∗^*p* < 0.001.

MF is often accompanied by myocardial hypertrophy, which manifests itself mainly as elevated CWI. As shown in Figure 1(c), the CWI of the MF mice was significantly elevated compared to that of the NC mice (*p* < 0.05), however, the overall CWI of the EA-treated mice was significantly lower than that of the MF mice (*p* < 0.05).

### 3.2. Rotarod Test

The mice in the MF group had shorter exercise endurance than the NC group. The EA group showed a tendency towards longer exercise, but the three groups were not statistically different (*p* > 0.05) (Figure 1(f)).

### 3.3. Effect of EA on Cardiac Function

Figure 1(a) shows typical ultrasound images of the three groups of mice. Statistical analysis of ultrasound parameters showed a decline in EF and FS in the MF group compared to the NC group (*p* < 0.05). EF and FS indices showed an upward trend in the EA group compared to the MF group (*p* < 0.05) (Figures 1(d) and 1(e)).

### 3.4. Effect of EA on the Change of cTnI

Troponin I is a useful marker of myocardial damage. It can be released from damaged myocardial cells into the blood when there is an external stimulus. Figure 1(g) shows that the serum concentration of cTnl was low in all mice in the three experimental groups. The EA group had significantly lower cTnl than the NC group (*p* < 0.05) and also less than the model group (*p* < 0.05).

### 3.5. EA Improves Myocardial Fiber Alignment Disorders

**Figure 2:**
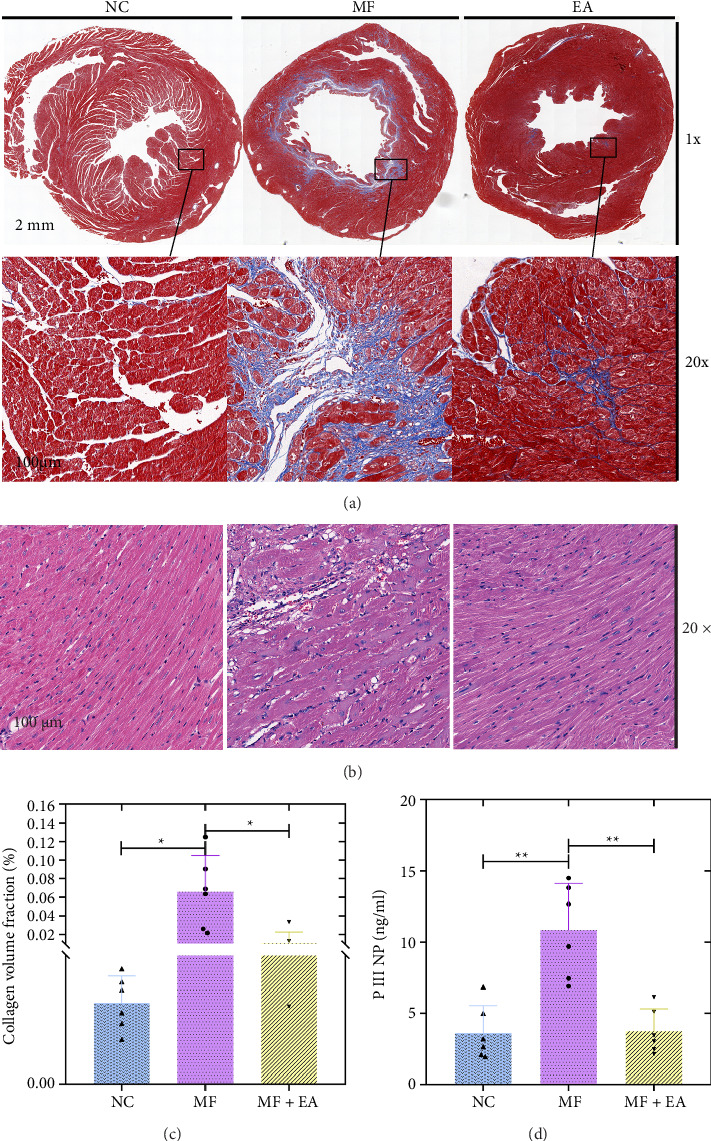
Electroacupuncture improves fibrosis in the myocardium. (a, b) Masson/HE staining of cardiac tissue (*n* = 6). (c) Collagen accumulation fraction. (d) PIIINP of mice (*n* = 6). ^∗^*p* < 0.05, ^∗∗^*p* < 0.01.

The cardiac tissues of the mice in the NC group did not show significant lesions, and the HE sections in the MF group showed infiltration of inflammatory cells and a disorganized arrangement of the cardiac myofibers. Mice in the EA group improved such pathological changes as described above (Figure 2(b)).

### 3.6. EA Reduces the Production of Collagen Fibers

Cardiac collagen fibrils can be observed through collagen Masson staining and analysis of serum PIIINP expression levels. Figures 2(a) and 2(c) show that Masson staining revealed the absence of collagen deposition in normal mice, whereas the MF group showed notable accumulation (*p* < 0.05) and the EA group showed restricted accumulation (*p* < 0.05). ELISA results (Figure 2(d)) showed that the MF group had higher PIIINP than the NC group (*p* < 0.05), while EA-treated mice had lower PIIINP (*p* < 0.05).

### 3.7. Effect of EA on ISO-Induced Intestinal Microorganism Imbalance

**Figure 3:**
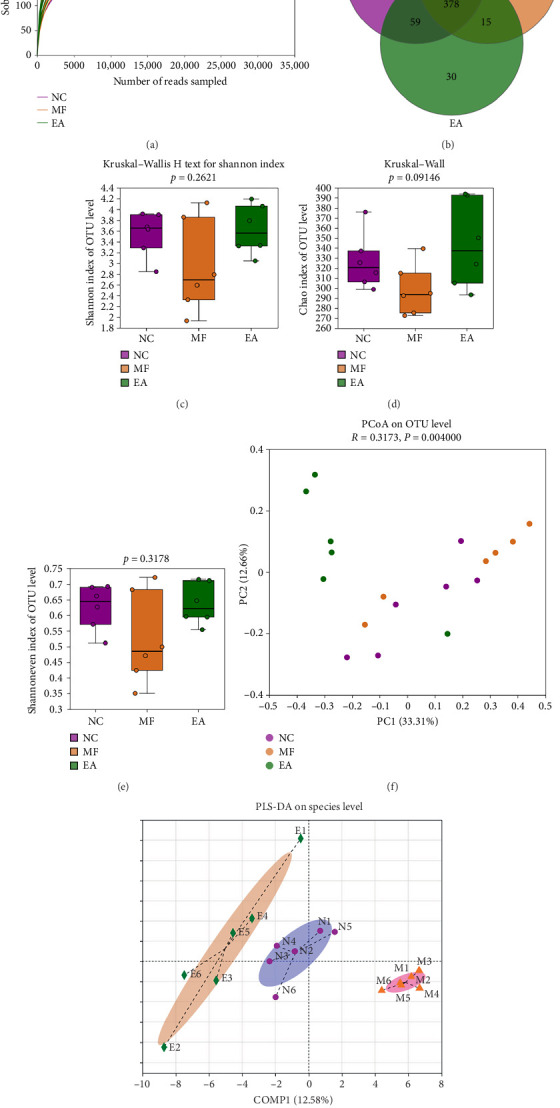
Electroacupuncture effect on the microbial structure of the gut of mice. (a) Sobs dilution curve. (b) Venn diagram of the OTU level of microorganisms. (c–e) Difference of alpha diversity among groups (Shannon/Chao/Shannoneven). (f, g) Principal coordinates analysis (PCoA) and partial least squares-discriminant analysis (PLS-DA).

519 OTUs were obtained from testing mice's faeces samples using 16S rDNA sequencing. The sequences from each sample were randomly assigned an identity sequence number, with the minimum number of sample sequences being the basis for this allocation. A dilution diagram with the extracted sequence and the corresponding OTUs (Figure 3(a)) showed a clear plateau period, indicating adequate sequencing.

### 3.8. Bacterial Colony Alpha and Beta Diversity Analysis

Samples from all groups were statistically calculated at the OUT level to plot a Venn diagram (Figure 3(b)). Additionally, 16 similar species were observed between the MF and NC groups, while 59 identical species were identified between the EA and NC groups. The results demonstrated that the abundance of the specified species increased following the EA intervention. Alpha diversity is defined as the diversity, abundance, and uniformity of microflora. As illustrated in Figures 3(c), 3(d), and 3(e), the Shannon, Chao, and Shannoneven indices of the faecal samples from the three groups demonstrated a reduction in microbial diversity, abundance, and uniformity following ISO treatment. The alpha diversity index of the faecal microflora of MF mice increased following EA therapy.

The principal coordinates analysis (PCoA) belongs to a nonbinding data dimensionality reduction method and is used to analyze the similarity or difference of samples. As shown in Figure 3(f), the samples in 3 groups separated significantly, which meant the difference in microbial structure among 3 groups was obvious (*R* = 0.3163, *p*=0.0040, the degree of contribution of PC1 and PC2 was 33.31% and 12.66%). Partial least squares-discriminant analysis (PLS-DA) is a supervised regression modeling method and is often used to distinguish the difference among groups. The microorganisms in 3 groups separated significantly, and it was macroscopic that the distance between the EA and NC groups was closer than that between the MF and NC groups (Figure 3(g)).

### 3.9. Bacterial Composition at Different Taxonomic Levels

**Figure 4:**
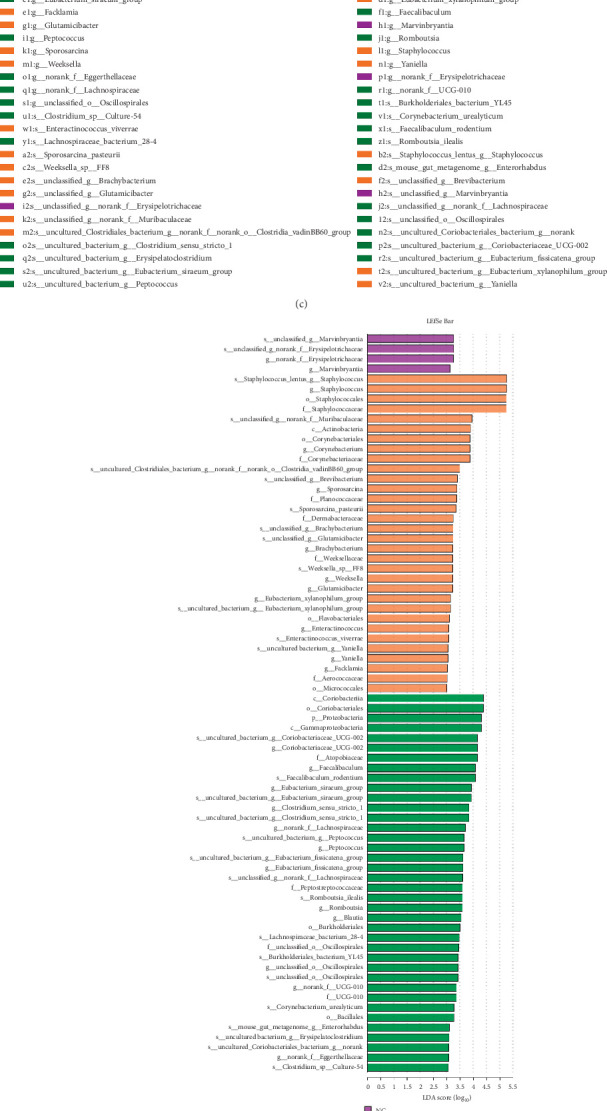
Electroacupuncture effect on the dominant intestinal flora of mice. (a, b) Relative abundance of gut microorganism at the level of phylum and species. (c, d) Cladogram and LDA score. (e) Species abundance with significant difference in 3 groups (best 15 abundance value). (f–h) Heatmap of the correlation between differential bacteria and biochemical indicators.

*Firmicutes*, *Bacteroidota*, *Verrucomicrobiota*, *Actinobacteriota,* and *Proteobacteria* were the main gut microflora of KM mice in the current experiment (Figure 4(a)). *Firmicutes* is the microflora with the highest abundance in all groups, and its abundance rate in the NC, EA and MF groups reached 46.20%, 36.48%, and 59.83%, respectively. The abundances of *Bacteroidota* were 50.44%, 42.11%, and 34.96%, respectively, in the NC, EA, and MF groups. Compared to the NC group, the relative abundance of *Firmicutes* microorganisms increased, but that of *Bacteroidota* decreased in the MF group, and EA therapy decreased Firmicutes abundance. Furthermore, the abundance of *Bacteroidota* microorganisms in the EA group increased compared to that in the MF group.

At the species level (Figure 4(b)), *the uncultured bacteria-g-norank-f-muribaculaceae* (*UM*)*, staphylococcus-lentus-g-staphylococcus* (*S. lentus*), *the uncultured bacteria-bacteroidales-g-Alloprevotella* (*u-Alloprevotella*) and *the uncultured bacteria-dales-g-norank-f-muribaculaceae* are the dominant microbiota species in the gut of KM mice. *UM* was the bacterium with the highest relative abundance rate in each group, and the rates reached 27.47%, 22.32%, and 18.79% in the NC, EA, and MF groups, respectively. Compared to the NC group, the relative microorganism abundance of *S. lentus* increased and that of *u-Alloprevotella* decreased in the MF group. Meanwhile, that of *S. lentus* decreased in the EA group. In addition, that of *u-Alloprevotella* in the EA group increased compared to the MF group. All data showed that EA therapy improved the structure of intestinal microorganisms which could be beneficial for the host.

Based on the abundance data of the flora at the species level in all groups, the species with a significant difference in the abundance of microorganisms between 3 groups performed a rank sum test statistic with Kruskal–Wallis, and the result of the 15 best species in the average abundance was plotted on a river map (Figure 4(e)). The abundance of *Muribaculaceae*, *Staphylococcus*, *Xylanophilum group,* and *Atopostipes* increased in the MF group, compared to the NC group, while the treatment with EA showed a decreasing trend. The abundance of flora of *Erysipelotrichaceae*, *Lachnospiraceae*, *Lachnospiraceae bacterium 28-4*, and *Clostridia UCG-014* decreased after ISO intervention but showed a rebound after EA therapy. The above results suggest that EA treatment can reduce the vast majority of species abundance alterations due to ISO.

Different bacteria communities in 3 groups were studied using the linear discriminant analysis effect size (LEfSe) and effect size of LDA > 3.0. As shown in the cladogram (Figure 4(c)), the difference in the dominant bacteria flora among the 3 groups was significant. LDA scores (Figure 4(d)) showed a predominance of *Staphylococcus lentus Staphylococcus*, *Staphylococcus*, *Staphylococcales* and *Staphylococcaceae* in the feces of ISO-treated mice. However, *Coriobacteriia*, *Coriobacteriales*, *Proteobacteria*, *Gammaproteobacteria*, and *Coriobacteriaceae UCG 002* were dominant after EA therapy.

### 3.10. Pearson Analysis of Differential Flora With Biochemical Indices

The 15 most abundant flora species, identified at the species level, were correlated with PIIINP, CWI, EF, FS, and CVF using Pearson's correlation analysis to generate the heat map.

The correlation between differential bacteria and collagen deposition indicators PIIINP and CVF is shown in Figure 4(f). PIIINP showed a significant positive correlation with the *Staphylococcus* and *Xylanophilum group* but a significant negative correlation with *Erysipelotrichaceae*. Meanwhile, CVF showed a significant negative correlation with *Erysipelotrichaceae*, while it also had a high positive correlation with the *Staphylococcus* and *Xylanophilum groups*.

Figure 4(g) shows the cardiac function indices EF and FS and the differential bacterial correlations. There is a significant positive correlation between EF/FS and *Lachnospiraceae*.

Figure 4(h) depicts the heat map of the correlation between the differential bacteria and the remaining indicators. Exercise tolerance exhibited a significant and positive correlation with *Stricto-1*. The CWI demonstrated a strong correlation with *Staphylococcus* and *Bacterium 28-4*. A significant negative correlation was observed between cTnl and *Coriobacteriaceae-UCG-002* and *Rodentium*.

### 3.11. Serum Metabolomics

#### 3.11.1. Hydrogen Spectra NMR Spectra

**Figure 5:**
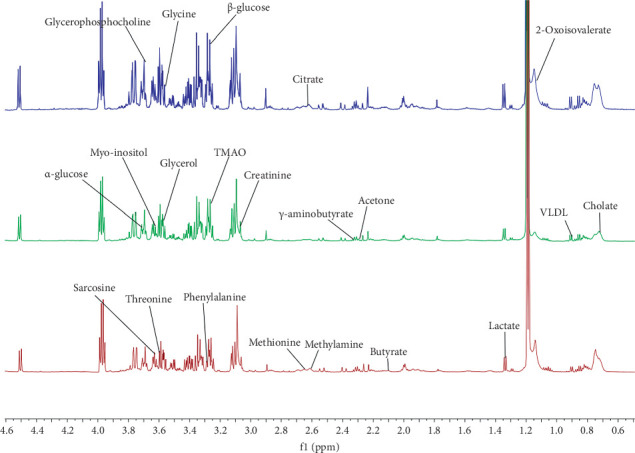
Hydrogen spectra NMR spectra.

NMR was used to obtain mouse serum spectra, as shown in Figure 5. The spectra data were exported for statistical analysis after being preprocessed with MestReNova. Finally, the metabolites selected, totaling 23, were identified based on existing literature studies, the Human Metabolome Database (HMDB), and the Kyoto Encyclopedia of Genes and Genomes (KEGG), and cumulative databases of previous studies of the subject group.

#### 3.11.2. Mice Serum Metabolite Profile Analysis

**Figure 6:**
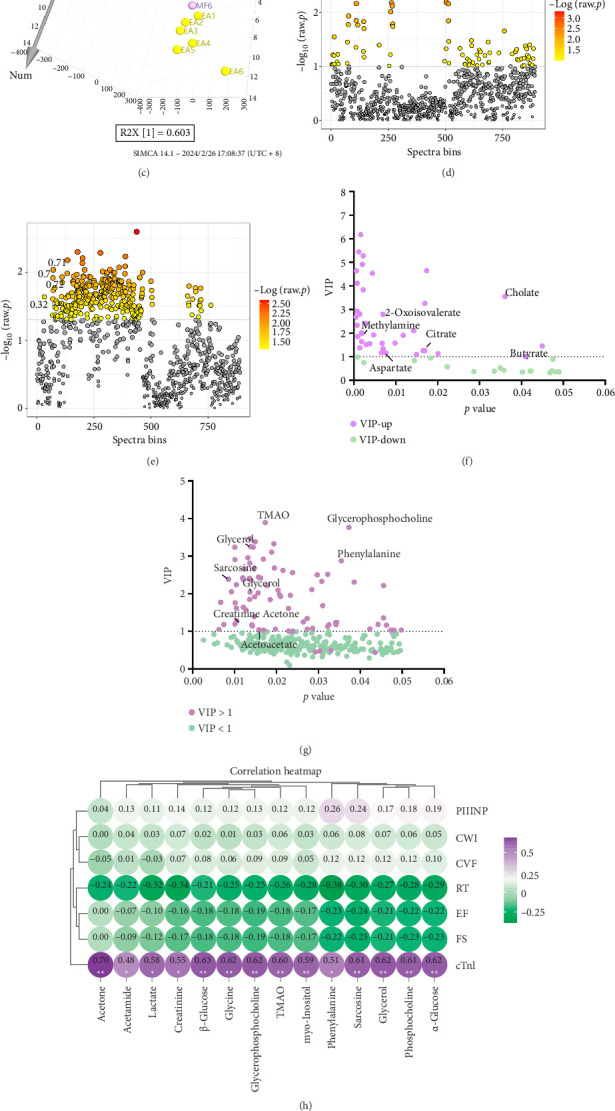
Electroacupuncture effect on serum metabolite of mice. (a) OPLS-DA of three groups. (b) OPLS-DA of the NC group and the MF group. (c) OPLS-DA of the MF group and EA group. (d) Significantly different chemical shifts of the NC group and the MF group. (e) Significantly different chemical shifts of the MF group and the EA group. (f) Differential metabolite of the NC group and the MF group. (g) Differential metabolite of the MF group and EA group. (h) Heatmap of the correlation between differential metabolite and biochemical indicators. (i) Heatmap of correlation between differential metabolite and differential bacteria.

Orthogonal PLS-DA (OPLS-DA) was used to explore differences in serum metabolite composition among three groups of mice. OPLS-DA (R2X = 0.71, R2Y = 0.358, *Q*2 = 0.136) revealed a trend of segregation of serum metabolite structures among the three groups of mice (Figure 6(a)).

#### 3.11.3. Differential Metabolite Analysis

In this study, serum metabolic data from the NC group, MF group, and EA group, respectively, were subjected to OPLS-DA analysis between groups to obtain the corresponding profiles. Significant separation of metabolites was found between the NC group and the MF group, which, in combination with the aforementioned expression of CWI, CVF, and PIIINP, suggests that ISO modeling affects serum metabolic profiles of mice (Figure 6(b)). As shown in Figure 6(c), comparing the MF group and the EA group, there was also a significant separation of metabolites between the groups, suggesting that the EA intervention could alter the serum metabolic profiles of the MF model mice.

The chemical changes were fragmented and integrated and then compared between the groups (Figures 6(d) and 6(e), *p* < 0.05). Next, statistically significant chemical changes were screened by combining the variable importance in the Projection (VIP > 1.0) of OPLS-DA as described above. Finally, specific metabolites were identified on the basis of chemical shifts and spectral peak patterns. The results showed that there were significant differences in the levels of five metabolites, including cholate and 2-oxoisovalerate, in the MF group compared to the NC group (Figure 6(f)). These aforementioned substances may be potential biomarker metabolites for MF. Meanwhile, the serum metabolic profiles of the EA group were compared with those of the MF group according to the equivalent method, and seventeen differential metabolites were screened, as shown in Figure 6(g).

### 3.12. Pearson Analysis of Differential Metabolites With Biochemical Indicators

The differential metabolites of the EA and MF groups were correlated with PIIINP, CWI, EF, FS, and CVF. As illustrated in Figure 6(h), cTnl demonstrated a markedly positive correlation with all differential metabolites. However, the remaining indicators exhibited no notable correlation with the metabolites.

### 3.13. Pearson Analysis of Microbiomics and Metabolomics

The 15 most notable strains and metabolites in EA were selected for Pearson analysis. The results obtained were presented in the form of heatmaps. As shown in Figure 6(i), which showed a significant negative correlation between the *Stricto-1* and Phenylalanine and *Rodentium* and acetone were significantly negatively correlated.

## 4. Discussion

### 4.1. Electroacupuncture Attenuates MF and Improves Cardiac Function

Cardiac diseases commonly involve excessive activation of myofibroblasts and ECM deposition, a pathogenesis linked to the RAAS activation. ISO activates β-adrenergic receptors, inducing tachycardia, hypotension, and cardiac overload. This stimulates the transformation of resting fibroblasts into collagen-producing myofibroblasts [18]. Collagen precursors are cleaved by collagenases, releasing protofibrillar collagen and propeptides (including PIIINP) into the ECM and bloodstream, respectively, ultimately leading to MF. Protofibrillar collagen types I and III alter the ECM, disrupt cardiac electrical conduction, and impair cardiac function. Concurrently, prolonged diastolic insufficiency, autonomic dysfunction, and myocardial ischemia contribute to myocardial hypertrophy and an increase in CWI.

In this study, Masson staining revealed significantly smaller areas of myocardial collagen deposition in EA-treated MF mice versus controls, indicating that EA reduces ECM deposition. This effect may involve inhibition of precollagen fiber production by myofibroblasts—a mechanism indirectly supported by serum PIIINP trends. EA further reduced fibrotic areas, enhanced cardiac compliance, and ameliorated myocardial hypertrophy, with these findings aligning with CWI, EF, and FS measurements.

Cardiac dysfunction impairs pulmonary function and reduces exercise tolerance. Clinically, as cardiac failure severity increases, 6 min walk test performance deteriorates [19]. Although the 14-day EA program improved exercise endurance, the small sample size (*n* = 6) precluded statistical significance. Free cTnl is released into the bloodstream from damaged cardiomyocytes following injury, serving as a standard biomarker for quantifying myocardial damage. Its concentration peaks on Day 1 postinjury and subsequently declines logarithmically [20]. In this experiment, cTnl sampling occurred on Day 11 post-ISO injection—during its decline phase. Nevertheless, EA intervention appeared to accelerate cTnl clearance.

### 4.2. Electroacupuncture Modulates Gut Microbiota Composition

The role of intestinal microorganisms and organism metabolites in human physiology aligns with the dynamic and holistic nature of organism regulation by traditional Chinese medicine acupuncture. The influence of gut microorganisms on CVD is mediated by downstream metabolites, including propionate, TMAO, SCFAs, and bile acids [21]. Conversely, CVD impairs cardiac output, causes tissue congestion, and induces vascular changes that directly compromise the intestinal barrier. This disruption triggers gut flora alterations and bacterial translocation, characterized by shifts in *Bacteroidota*, *Firmicutes*, and *Proteobacteria* abundances. In this study, EA primarily modulated the intestinal *Bacteroidota*/*Firmicutes* ratio in MF mice.

#### 4.2.1. Beneficial Effects of Increased *Bacteroidota*

Many studies have suggested that the level of *Bacteroides* in the intestine is negatively correlated with the severity of CVD [22], which is consistent with the results of the present experiment in which the ratio of *Bacteroidota* in the intestine of MF mice decreased. A decrease in the content of the bacterium *Bacteroidota* is often accompanied by a decrease in EF and FS in patients with type 2 diabetes [23]. This finding has led to the hypothesis that a reduction in *Bacteroidota* may contribute to an increase in the thickness of the left atrium, thereby increasing the risk of structural cardiac disease. This process may be associated with inflammation and oxidative stress [24]. Naofumi Yoshida experimentally demonstrated that *Bacteroidota* tube feeding reduces the effects of atherosclerosis in mice, which is closely related to changes in fecal and plasma lipopolysaccharide levels [25]. The cardioprotective effects of *Bacteroidota* may be related to biometabolic functions.

The *Bacteroidota* are primarily engaged in the fermentation of carbohydrates, the utilization of nitrogen-containing substances, and the bioavailability of bile acids and other steroids [26]. These processes, in turn, exert a regulatory influence on CVDs through the signalling functions of products such as propionate, acetate, and secondary bile acids generated by metabolism. Propionate has been demonstrated to regulate blood pressure by acting on Olfr78 receptors, thereby modulating renin release and diastolic vascular smooth muscle [27]. Additionally, propionate has been shown to modulate T-lymphocyte-mediated inflammation, which in turn slows vascular dysfunction and MF [21]. Acetate vasodilates blood vessels through parasympathetic nerves, downregulates heart rate, exerts negative inotropic effects on the heart, and reduces host cardiac prevalence [28–30]. In this experiment, the proportion of beneficial bacteria *Bacteroidota* in the intestinal tract of EA-treated MF mice increased.

#### 4.2.2. Detrimental Effects of Increased *Firmicutes*

More *Firmicutes* bacteria in the intestines can harm the heart. LiCui [31] compared faecal samples from patients with coronary artery disease and healthy individuals. The results showed elevated levels of *Firmicutes* bacteria in the former. And *Firmicutes* were enriched in apolipoprotein E knockout mice raised on a Western diet [32], which is in line with the present experimental observation of elevated *Firmicutes* levels in the intestines of MF mice. In addition, Ruijing Yan found that cardiac inflammation was often accompanied by elevated ratios of *Proteobacteria* and *Firmicutes* [33]. This may be related to the fact that upregulation of *Firmicutes* content leads to increased levels of lipopolysaccharides in the host, which can promote the development of chronic inflammation and exacerbate the development of CVD. In this experiment, the proportion of *Firmicutes* in the intestinal tract of EA-treated MF mice decreased.

### 4.3. Correlations Linking Cardiac Markers, Gut Microbiota, and Exercise Tolerance

PIIINP and CVF were positively correlated with the *Staphylococcus lentus group* and *Xylanophilum group*. *Staphylococcus lentus* is a Gram-positive bacterium belonging to the genus *Staphylococcus*, which is closely related to fibrosis. It has been demonstrated that the administration of *Staphylococcus lentus* via tube feeding can precipitate an exacerbation of the biochemical indices and pathological manifestations of liver fibrosis in mice. This may be attributable to an increased intestinal permeability and an inflammatory response induced by *Staphylococcus lentus* [34]. *Staphylococcus* spp. have a proinflammatory effect on the gut and are positively correlated with high-sensitivity CRP, an indicator of inflammation in coronary syndromes [35]. *The Xylanophilum group* belongs to *Eubacterium*, the same Gram-positive bacterium that is one of the core genera of the human intestinal tract and is associated with the development of CVD. In the intestinal flora of rats with a deoxycorticosterone acetate-induced hypertension model, the *Eubacterium* ratio was elevated and was accompanied by fibrosis and hypertrophy of the heart and kidneys [36]. Another clinical case noted that *Eubacterium* was detected in the pericardial effusion of patients with right heart failure [37]. In diabetic patients treated with a combination of probiotics, blood glucose and lipids were decreased along with *Eubacterium* in the gut [38].

PIIINP and CVF were negatively correlated with *Erysipelotrichaceae*. *Erysipelotrichaceae* is a highly immunogenic bacterium, capable of activating immune cells and eliciting an immune response [39]. This may be associated with recovery from CVD, although there is no definitive evidence of a direct relationship between this bacterium and CVD.

EF and FS showed a positive correlation with *Lachnospiraceae*. A positive correlation was observed between EF, FS, and *Lachnospiraceae*. The distribution of *Lachnospiraceae* is diminished in the intestine of patients with heart failure in comparison to that observed in normal subjects. Nevertheless, following cardiac rehabilitation, a distinct correlation was observed between the recuperation of cardiac function and the presence of this bacterium [40].

Exercise tolerance was significantly and positively correlated with *Stricto-1 bacteria*. There are fewer studies on this bacterium, and there is no clear evidence of a correlation with CVD. However, *Stricto-1* belongs to *Bacteroidota*, which are mostly beneficial intestinal bacteria that play a role in cardiovascular protection.

### 4.4. Electroacupuncture Effects on Metabolites and Underlying Mechanisms in Mice

In this experimental work, we also tested the effect of EA on metabolomics in MF mice. The results showed that TMAO, lactate, creatinine, creatine, acetone, glycose, glycine, glucose, choline phosphate, and glycerophosphorylcholine were the differential metabolites after treatment with EA.

There is a close link between lipid metabolism and CVD. Abnormal metabolism of glycerophospholipids induces metabolic and CVDs [41], while phosphatidylcholine, phosphatidylethanolamine, and phosphatidylserine account for the highest percentage of glycerophospholipids. Phosphocholine-modified macromolecules have been demonstrated to be effective in inhibiting the ATP-dependent release of interleukin-1β and in suppressing inflammatory responses. This is achieved through a mechanism involving nicotinic acetylcholine receptors [42, 43].

Glycerol is a metabolic substrate that is esterified in fatty acids to form triglycerides. Although triglycerides are important energy-supplying and energy-storing substances in the human body, elevated levels of triglycerides may be associated with a series of metabolic diseases such as atherosclerosis and obesity, which can increase the risk of heart disease. Three pathways have been identified for the synthesis of TMAO: choline, lecithin, and L-carnitine-rich dietary matrices such as eggs, milk, and liver, which are catabolized by *Clostridium*, *Enterobacteriaceae*, and *Fusobacteriaceae* in the host intestine to synthesize TMA, and then finally oxidized to TMAO in the liver [44, 45]. The specific metabolic pathway is that dietary choline is cleaved to TMA by choline-TMA lyase-containing *Sclerotinia*, *Proteobacteria*, and *Actinobacteria*, and TMA is recycled by the portal system into the liver, where it is oxidized by liver flavin monooxygenase to TMAO. TMAO causes a decrease in Cyp7a1 and an increase in CD36, SR-A1, COX2, IL-6, ICAM1, IL-18, and IL-1β by affecting the MAPK/NF-κB and SIRT3-SOD2-mtROS pathways [46]. These cause unfavorable biological responses affecting the heart, including decreased reverse cholesterol transport, increased thrombotic risk, and the activation of vascular inflammation and the immune system. Creatinine is metabolized by both endogenous and exogenous pathways, the metabolism of meat foods and native muscle, respectively. Patients with underlying heart disease have higher levels of creatinine compared to the normal population [47]. Phenylalanine is an aromatic amino acid that is nutritionally essential. Its normal metabolic pathway is through hydroxylation by phenylalanine hydroxylase to generate tyrosine, which is then metabolized by hydroxylation and other metabolic pathways to catecholamine neurotransmitters, including dopamine, norepinephrine, and epinephrine. Catecholamine neurotransmitters typically exert a cardiac excitatory effect and are not conducive to repairing cardiac injury. Lactate is converted by lactate dehydrogenase from pyruvate to the end product of anaerobic or aerobic glycolysis. Furthermore, mature adipocyte lactate is associated with metabolic disorders such as diabetes, and it has been demonstrated to elevate the level of IL-1β in macrophages and promote adipose tissue inflammation [48].

### 4.5. Association Analysis: Differential Microbiota and Metabolites

Microbiomic and metabolomics correlation analyzes showed a significant negative correlation between *Stricto-1* and Phenylalanine content, and a significant negative correlation between *Rodentium* and acetone content. Both bacteria belong to the *Bacteroidota* genus, which plays a beneficial role in cardiovascular health. *Bacteroidota* act on GPR43, OLFR78, MUC2, and GPR41 through the signalling function of products generated by metabolism, such as propionate, acetate, and secondary bile acids, thereby regulating blood pressure and protecting the cardiovascular system through insulin-mediated mechanisms [49].

Acetone and phenylalanine metabolites are not cardiovascularly favorable. The heart converts energy metabolism to increased utilization of ketone bodies in failure, and acetone, a product of ketone metabolism, is elevated in both blood and urine in patients with heart failure [50]. And exhaled acetone has also been used in recent years as a new biomarker to aid in the diagnosis of heart failure and is elevated as the degree of heart failure increases [51]. The mechanism for the elevation of ketoacids in patients with heart failure is unclear, but it may be related to fat metabolism induced by high levels of circulatory stress in the heart, leading to an increase in free fatty acids [52]. Phenylalanine undergoes hydroxylation and metabolism to tyrosine, which is then metabolized into catecholamine neurotransmitters such as dopamine, norepinephrine, and epinephrine, which activate calcium-sensing receptors to induce pulmonary hypertension [53] and also play an important role in cardiac senescence [54]. The results of this experiment showed a decreasing trend for both acetone and phenylalanine and an increasing trend for *Bacteroidota*, and the correlation results were consistent with previous studies [55].

## 5. Conclusion

This study demonstrates that EA effectively ameliorates MF in mice. EA intervention modulated the intestinal microbiota by increasing the abundance of *Bacteroidot*a and decreasing the abundance of *Firmicutes*. Significant correlations were observed between specific differential bacteria and key disease indices. Furthermore, EA reduced the levels of detrimental metabolites, including acetone, phenylalanine, and TMAO, which themselves correlated with the altered microbiota. Collectively, these findings suggest that the therapeutic efficacy of EA in improving MF may be mediated through its regulation of the gut microbiota and associated host metabolic pathways.

## Data Availability

The datasets generated and analyzed during the current study are available from the corresponding author upon reasonable request.
